# Novel neurobiological roles of UBE3A

**DOI:** 10.18632/oncotarget.15105

**Published:** 2017-02-05

**Authors:** Jiandong Sun, Michel Baudry, Xiaoning Bi

**Affiliations:** Western University of Health Sciences, Pomona, CA, USA

**Keywords:** Angelman syndrome, mTORC1, synaptic plasticity, UBE3A, ubiquitination, Neuroscience

The mechanistic target of rapamycin (mTOR) is a highly conserved and ubiquitously expressed kinase, which consists of two distinct protein complexes, referred to as mTOR complex 1 (mTORC1) and mTOR complex 2 (mTORC2). mTORC1, coupled to Raptor, and mTORC2, coupled to Rictor, respond to different signals and target different substrates; while mTORC1 is activated by a number of signals, including growth factors, amino acids, oxygen, and energy status, mTORC2 activation is not well characterized [1]. Both mTORC1 and mTORC2 play critical roles in brain development and synaptic plasticity [2], although much less is known regarding the roles of mTORC2. Emerging evidence also implicates abnormal activation of mTOR pathways in multiple monogenetic neurodevelopmental disorders linked to autism spectrum disorders (ASD).

UBE3A, aka E6-associated protein (E6AP), the founding member of the HECT (homologous to E6AP carboxy terminus) domain-containing E3 ligase family [3], plays important roles in brain development and function, as UBE3A deficiency results in Angelman syndrome (AS) [4], while UBE3A over-expression increases the risk for autism [5]. Using an AS mouse model, we have shown that imbalanced signaling of the mTOR pathway, with increased mTORC1 and decreased mTORC2 activation, is critically involved not only in UBE3A deficiency-induced cerebellum-dependent motor dysfunction [6], but also in hippocampal synaptic plasticity and fear-conditioning memory deficits [7]. The mTORC1 inhibitor, rapamycin, S6K1 inhibitor, PF-4708671, or mTORC2 activator, A-443654, all rescued hippocampal long-term potentiation (LTP) and actin polymerization in field CA1 of hippocampus in AS mice. Since both rapamycin and PF-4708671 treatments reversed the decrease in mTORC2 activity in AS mice, it is likely that mTORC1-S6K1 over-activation is responsible for reduced mTORC2 activation in AS mice.

**Figure 1:**
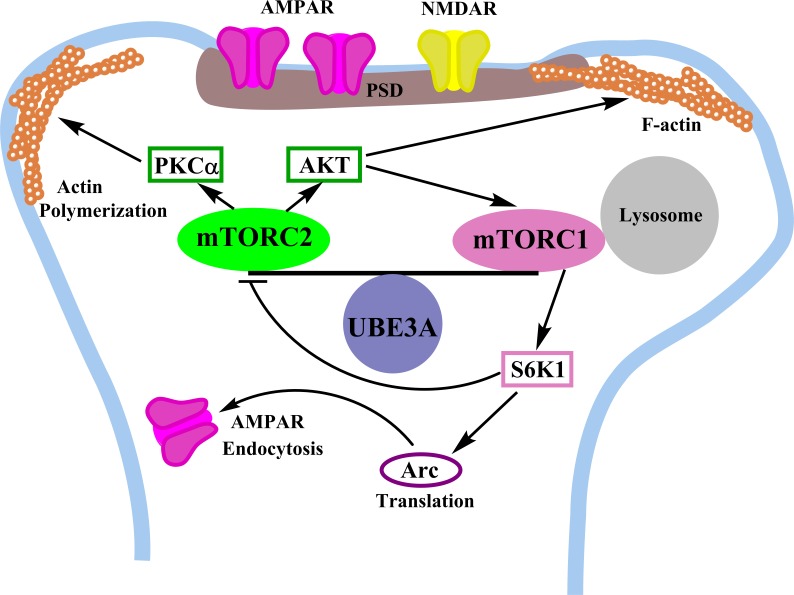
Roles of UBE3A in the regulation of mTORC1 and mTORC2 signaling

Our results showed that enhanced mTORC1-S6K1 activities may contribute to LTP and learning impairment in AS mice through increasing local protein synthesis, including that of Arc, which then stimulates AMPA receptor endocytosis (Figure 1). Our results also provided evidence that mTORC2 plays an important role in maintaining spine morphology through the regulation of cytoskeletal network, either directly or indirectly via PKCα and other downstream effectors.

Recent research has identified a number of potential UBE3A substrates with diverse functions. A future challenge consists in understanding the underlying molecular basis for AS by putting together the currently available data from both animal and human studies. Identifying convergent molecular pathways in which multiple candidate substrates are involved constitutes a promising start. Recent studies have suggested that AS pathophysiology may originate from abnormal activity-dependent neuronal development and synaptic plasticity. Given the critical roles of mTOR signaling network in brain development and function, its dysregulation in AS may be a guiding principle. Notably, on the basis of clear links between enhanced mTOR signaling and autism, the mTOR pathway represents a promising therapeutic target for the treatment of ASDs.

How UBE3A deficiency results in mTORC1 over-activation remains to be elucidated. In this regard, accumulating evidence indicates that a lysosome-based signaling system composed of Rag GTPases, Ragulator, v-ATPase, and GATOR, is crucial for mTORC1 activation [8]. The mechanisms involved in amino acid sensing and mTORC1 signaling are starting to be understood, and multiple amino acid sensors (e.g., SLC38A9, CASTOR1, and Sestrin2) have been identified. Although regulation of mTORC1 by the TSC complex-Rheb axis is well documented in brain, the amino acid branch has rarely been studied. Whether UBE3A regulates mTORC1 activation by ubiquitination of some components of the amino acid branch is an intriguing question. Better understanding of this pathway should shed new light on basic neurobiological mechanisms as well as on several neurological and neuropsychiatric diseases.
